# Prevention of Sexual Diseases

**Published:** 1911-02

**Authors:** 


**Prevention of Sexual Diseases.** By **Victor G. Vecki,** M.D., San Francisco. With introduction by **William J. Robinson,** M.D. 12 mo, pp. 132. New York: Gritic and Guide Co. (Cloth, $1.50.)

This excellent little book discusses sexual prophylaxis from every point of view. The text is outspoken although commendably free from unclean language. The sanitary regulations of prostitution is advocated, the author claiming that venereal disease is not as prevalent in communities where inspection is properly carried out. Alcoholism is considered a marked etiologic factor, stimulating licentiousness and increasing susceptibility. Antiseptic post coital measures are recommended, an instance being cited where 256 sailors were treated after exposure and all escaped infection. C. W. B.

